# Supplementary material for: Displacement of Hospital-Acquired, Methicillin-Resistant Staphylococcus aureus Clones by Heterogeneous Community Strains in Kenya over a 13-Year Period
Source: Microorganisms. 2024 Jun 8;12(6):1171. doi: 10.3390/microorganisms12061171 (PMC11205442; doi:10.3390/microorganisms12061171)
Supplement: Supplementary file 1 [file microorganisms-12-01171-s001.zip › microorganisms-2948232 -Supplementar.pdf]

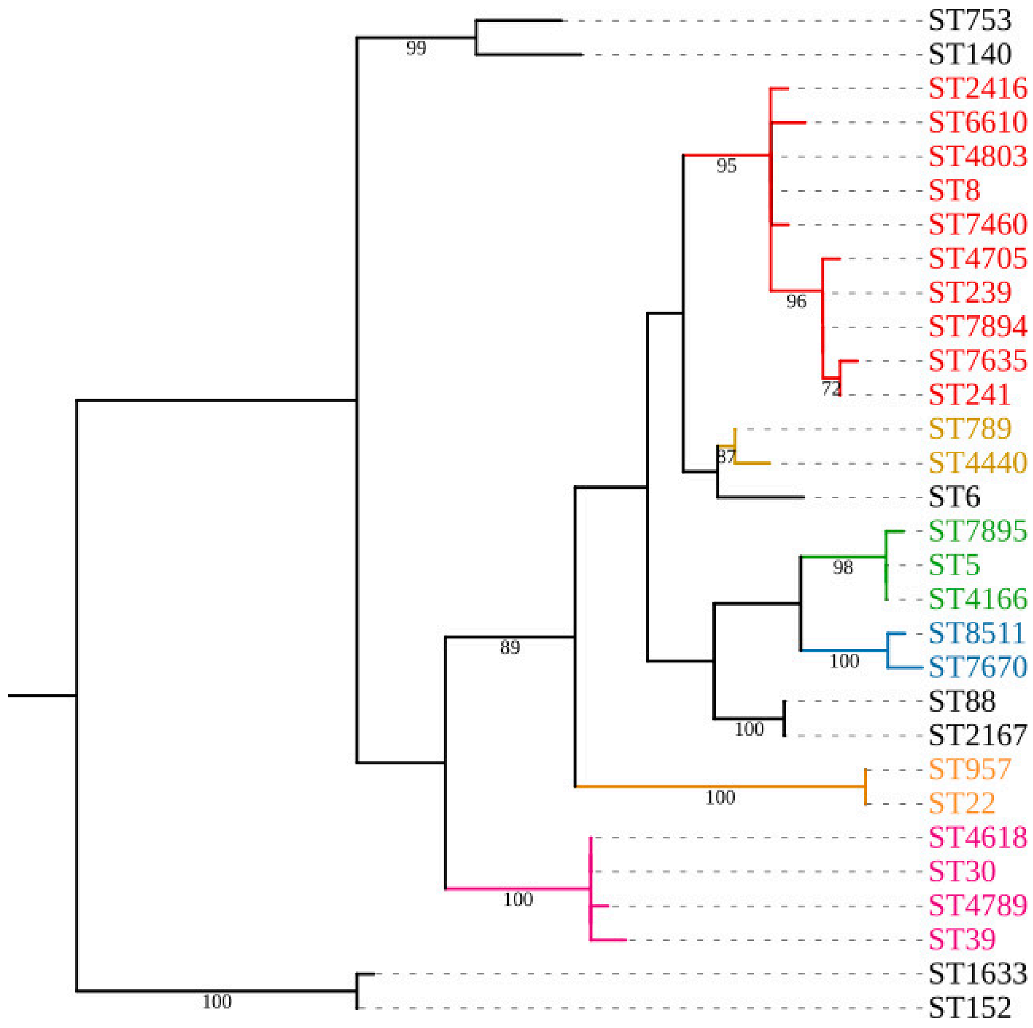

CC	*spa* type	SCC*mec*	Frequency	2010-17	2018-23
**N/A**	t011	V	1	-	+
**N/A**	N/A	IV	1	+	-
**CC8**	t1476	IV	2	-	+
**CC8**	t293	IV	2	+	-
**CC8**	t008/t024/t064/t1476	IV/V	30	+	+
**CC8**	t1476/t104	IV	5	+	+
**CC8**	t1476	IV	2	-	+
**CC8**	t2029	III	1	+	-
**CC8**	t037	III	6	+	-
**CC8**	t037/t11766	III	6	+	-
**CC8**	t030/t037	III	4	+	+
**CC8**	t037/t2029	III/IV	23	+	-
**CC7**	t091	V	1	+	-
**N/A**	t091	V	1	-	+
**N/A**	t304/t648	IV	4	+	+
**CC5**	t002	II	1	-	+
**CC5**	t13150	II	1	+	-
**CC5**	t442/t6100	IV	2	+	+
**CC1**	t127	IV	2	-	+
**N/A**	t657/t345	V	3	+	-
**N/A**	t1339	N/A	1	+	-
**N/A**	t1855/t16503	IV	4	-	+
**CC22**	t223/t852/t005	IV	3	+	-
**CC22**	t005/t022	IV	3	+	-
**CC30**	t318	IV	5	+	+
**CC30**	t318	IV	2	+	+
**CC30**	t964	IV	1	-	+
**CC30**	t007	II	1	+	-
**N/A**	t355	IV	3	+	+
**N/A**	t355/t8987/t11541	IV	14	+	+

Figure S1: A midpoint-rooted MLST phylogenetic tree of 136 MRSA strains. The tree was constructed using MEGA Version 11.0.11 based on 1000 bootstrap iterations and refined using the Interactive Tree of Life (iTOL) Version 5. **CC:** Clonal complex; **(+)** denotes strains reported in the indicated period while **(-)** denotes absence. Strains that form singletons are indicated as **N/A** on the CC column. Missing *Spa* and SCC*mec* types are indicated with N/A in the respective columns. Nodes with bootstraps support values >70% are indicated.

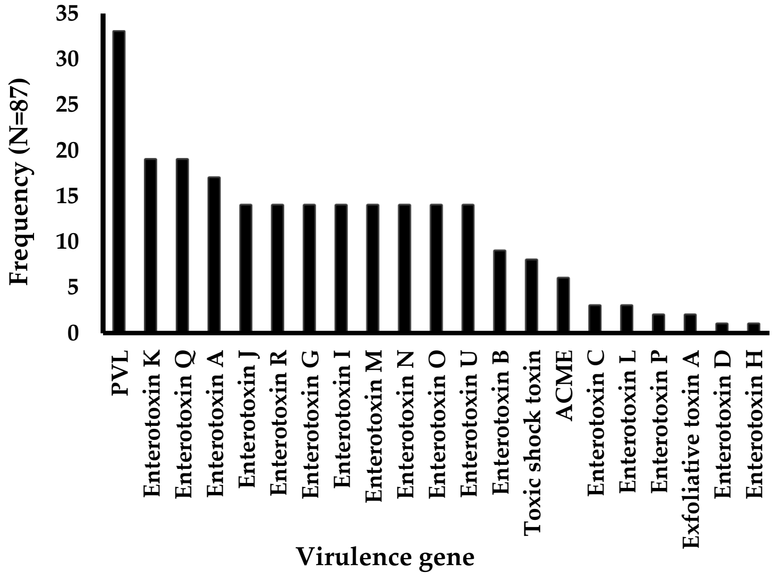

Figure S2: Summary of toxin gene carriage among eighty-seven MRSA genomes. ACME: Arginine catabolic mobile element; PVL: Panton-Valentine Leucocidin.

Table S1: Antimicrobial efflux and other resistance determinants

Resistance determinant	Antimicrobial class	Mechanism	Prevalence (%), (N=87)
norA	Fluoroquinolone, disinfectants & antiseptics	MFS efflux	15
norC	Fluoroquinolone, disinfectants & antiseptics	MFS efflux	40
sdrM	Fluoroquinolone, disinfectants & antiseptics	MFS efflux	91
mgrA	Fluoroquinolones, tetracyclines, cephalosporins, penams, disinfectants, antiseptics	ABC/MFS efflux	93
lmrS	Macrolides, aminoglycosides, folates, phenicols, oxazolidinones	MFS efflux	82
kdpD	Aminoglycosides	Antibiotic efflux	15
mepA	Tetracyclines, glycylcyclines	MATE efflux	89
tet38	Tetracyclines	MFS efflux	97
qacJ	Disinfectants & antiseptics	SMR efflux	14
qacG	Disinfectants & antiseptics	SMR efflux	1
sepA	Disinfectants & antiseptics	SMR efflux	78
fosB	Phosphonic acid antibiotics	Drug inactivation	54
GlpT [F3I, A100V]	Phosphonic acid antibiotics	Reduced uptake	11
MurA [D278E, E291D]	Phosphonic acid antibiotics	Target alteration	15
MurA [G257D]	Phosphonic acid antibiotics	Target alteration	21

ABC: ATP-binding cassette; **MATE:** Multidrug and toxic compound extrusion; **MFS:** Major facilitator superfamily; **SMR:** Small multidrug resistance pumps
